# Dendritic Cells/Macrophages-Targeting Feature of Ebola Glycoprotein and its Potential as Immunological Facilitator for Antiviral Vaccine Approach

**DOI:** 10.3390/microorganisms7100402

**Published:** 2019-09-29

**Authors:** Titus Abiola Olukitibi, Zhujun Ao, Mona Mahmoudi, Gary A. Kobinger, Xiaojian Yao

**Affiliations:** 1 Laboratory of Molecular Human Retrovirology, Department of Medical Microbiology, Max Rady College of Medicine, Rady Faculty of Health Sciences, University of Manitoba, Winnipeg, MB R3E 0J9, Canada; olukitit@myumanitoba.ca (T.A.O.); zhujun.ao@umanitoba.ca (Z.A.); mahmoud1@myumanitoba.ca (M.M.); 2 Centre de Recherche en Infectiologie de l’ Université Laval/Centre Hospitalier de l’ Université Laval (CHUL), Québec, QC G1V 4G2, Canada; Gary.Kobinger@crchudequebec.ulaval.ca

**Keywords:** Ebola glycoprotein, antiviral vaccine, immune response, dendritic cells and macrophages

## Abstract

In the prevention of epidemic and pandemic viral infection, the use of the antiviral vaccine has been the most successful biotechnological and biomedical approach. In recent times, vaccine development studies have focused on recruiting and targeting immunogens to dendritic cells (DCs) and macrophages to induce innate and adaptive immune responses. Interestingly, Ebola virus (EBOV) glycoprotein (GP) has a strong binding affinity with DCs and macrophages. Shreds of evidence have also shown that the interaction between EBOV GP with DCs and macrophages leads to massive recruitment of DCs and macrophages capable of regulating innate and adaptive immune responses. Therefore, studies for the development of vaccine can utilize the affinity between EBOV GP and DCs/macrophages as a novel immunological approach to induce both innate and acquired immune responses. In this review, we will discuss the unique features of EBOV GP to target the DC, and its potential to elicit strong immune responses while targeting DCs/macrophages. This review hopes to suggest and stimulate thoughts of developing a stronger and effective DC-targeting vaccine for diverse virus infection using EBOV GP.

## 1. Introduction

### 1.1. Dendritic cell (DC)-targeting Vaccines

The development of the antiviral vaccine has been the most successful biotechnological and biomedical approach against epidemic and pandemic viral infections [1]. Qualities of an ideal vaccine include safety (even in an immunocompromised patient), high effectivity in inducing immune responses [2], cost effectivity, and high stability and durability state [2]. There have been successful productions of many antiviral vaccines using different strategies, including live attenuated vaccines for yellow fever, smallpox, poliovirus, measles, mumps, rubella, adenovirus, varicella, and rotavirus; inactivated vaccines for poliovirus, influenza virus, hepatitis A virus, Japanese encephalitis; and virus-like particle (VLP) vaccines for hepatitis B and human papillomavirus (Table 1) [1,3]. In recent years, the impact of the new advent of technology in gene delivery and expression, adjuvants, the convergence of human monoclonal antibody isolation, structural biology, and high throughput sequencing, among others, has greatly influenced biotechnological approach for the development of new vaccines [4].

Despite the progress that has been recorded so far in vaccine development for viral infection, limitations such as the narrow-spectrum effect of vaccine and low immune response call for a new approach in the event of vaccines development [26]. Attention has thus been shifted to the abilities of dendritic cells (DCs)/macrophages to induce potent immune responses [27], and their usage is in the pipeline for the development of vaccines against cancer [28], adenovirus [29], and yellow fever [30], among others. A DC-targeting vaccine approach depends on the ability of DCs to target specific antigens by recognizing pathogen-associated molecular patterns (PAMPs) of the antigen, and further stimulate innate, humoral, and corresponding cellular immune responses [31]. Hawiger et al. showed that an antigen delivery system targeting the DEC-205, which is a DC-restricted endocytic receptor, using monoclonal antibody of DC induced a high magnitude of T cell responses [32]. Zaneti et al. also demonstrated recently that a DNA vaccine consisting of plasmid encoding single-chain Fv antibody (ScFv) αDEC205 fused with dengue virus (DENV) envelope domain III (EDIII) induced a strong anti-EDIII IgG titer and CD4^+^ capable of inhibiting DENV2 infection when intramuscularly injected into Balb/c mice followed by electroporation [33]. Table 2 shows a summary of other different strategies that have been used to target DCs for the development of vaccines.

However, there are some limitations. For instance, Cheng et al. showed that the mechanism of targeting DCs using recombinant adenovirus (rAd) vector vaccine is associated with toxicity related to ‘prior human gene therapy fatality’ [34]. Also, a study by Boudewijns et al. revealed the toxicity profile associated with DC vaccination in stage III and stage IV melanoma patients. In this case, melanoma patients were vaccinated with DCs loaded with antigens associated with the tumor. About 84% of the patients had adverse events related to the treatment, including reaction at the injection site, and symptoms such as flu, while about 3% of the patients experienced grade 3 toxicity [35]. However, some of the adverse effects noted are considered to be normal clinical or immunological responses [35].

Hawiger et al. also showed that the T cell activated by DC-targeted antigen could not be polarized to produce T helper cells. Hawiger et al. further demonstrated that T cells severely reduced after seven days and were not responsive to systemic antigen challenge [32]. Almand et al. also confirmed that the production of immature myeloid cells might cause dysfunction of DCs and can lead to immunosuppression of T cells [36]. However, Apostolico et al. demonstrated the induction of long-lived T cells against HIV using a DC-targeting approach with a heavy-chain αDEC205 (αDECHIVBr8) in the presence of TLR3 agonist [37].

### 1.2. Dendritic Cells (DCs)/Macrophages and Immune Responses

DCs are antigen-presenting cells (APCs) capable of initiating and directing innate and adaptive immune responses [44]. The intricate properties of DCs that account for their roles in the immune system are: Unique mechanisms for antigen presentation, the ability to migrate to a particular site in lymphoid organs for immune response stimulation, and their rapid differentiation or maturation in response to a variety of stimuli ranging from Toll-like receptor (TLR) ligands to many other non-microbial factors [45]. Briefly, after exposure to the foreign material, the DCs mature and migrate to the lymphoid organ, where the DCs induce a cellular immune response (T cells) and humoral immune responses (B cells) [46]. Targeting peptides to DCs can also induce an innate immune response by activating natural killer cells and natural killer T cells [47]. DCs also function by producing protective cytokines—like interleukin (IL)-12, IL-6 [48], and type I interferons [49], which influence distinct steps in the adaptive immune response of lymphocytes—and the activation and expansion of innate lymphocytes [45,49,50].

The presence of specialized surface receptors, known as pattern recognition receptors (PRRs), on DCs facilitate the functions of DCs. These PRRs are named as follows: Toll-like receptors (TLRs), NOD (Nucleotide-binding oligomerization domain)-like receptors (NLRs), C-lectin type receptors (CLRs), RIG-1 like receptors (RLRs), and helicases recognize pathogens associated molecular patterns (PAMPS) [51,52]. DCs play an essential role in conferring protection against pathogens and commensal microorganisms [53].

The TLRs, known as ‘sensors that detect infection’, were the first discovered PRRs [54]. TLRs are innate immune receptors with a full length of a membrane that can use pattern recognition processing of ligands to detect a variety of molecules that insinuate tissue damage, and a wide range of human pathogens including bacteria, viruses, protozoans, and nematodes [54,55]. The conserved pathogen recognition features of TLRs have led to the stimulation of several immune cells, not excluding proinflammatory cytokines, antimicrobial molecules, phagosomal maturation, and costimulatory molecules [56]. There are thirteen (13) known TLRs that can recognize a wide range of microbial pathogens, but differ in their specificity for microbial patterns. For instance, to recognize microbial cell walls and membranes unique to pathogens, TLRs 1, 2, 4, 5, and 6 are much employed; TLR4 recognizes lipopolysaccharides (LPS), while heterodimers of TLR2/1 and TLR2/6 recognize lipopeptides and TLR5 recognizes flagellin; TLR9 recognizes DNA unmethylated CpG motifs, various forms of RNA by TLRs 3, 7, 8 and 13; and TLR11 recognizes profilin and flagellin of *Salmonella.* Additionally, Fukuda et al. demonstrated that TLR9 that has an affinity for bacterial DNA ligands plays a crucial role by activating proinflammatory cytokines of macrophages, leading to the development of vascular inflammation and atherogenesis [57], while Koblansky et al. reported that the previously uncharacterized TLR12 can recognize *Toxoplasma gondii* profilin by plasmacytoid dendritic cells (pDCs) [58]. TLRs are localized intracellularly. As already reviewed, TLRs 3, 4, 7, and 9 have their transmembrane domain localized intracellularly [59]. Also, Raetz et al. reported that TLR11 and TLR12 are both intracellularly localized, where they both bind with *T. gondii* and lead to the signaling of MyD88- and UNC93B1-dependent signaling cascade [60].

Importantly, TLRs coordinate both the innate and adaptive immune responses [54,55,61]. Innate immune responses are activated via recognition of microbial products by TLRs, leading to the stimulation of proinflammatory cytokines maturation of DCs for antigen presentation. Also, the activation of DCs via TLRs can increase the level of proinflammatory cytokines, chemokines, and co-stimulatory molecules produced, thus modulating adaptive immune responses, including T regulatory cells [61]. More importantly, TLR4 can also recognize the EBOV glycoprotein (GP). Okumura et al. revealed that the sensor for EBOV GP is the host TLR4, which leads to the production of proinflammatory cytokines. Their study proved that EBOV GP could stimulate the expression NF-κB in vitro in a TLR4-dependent manner [62]. Moreover, Lai et al. pretreated mice with TLR4 antagonist (ultrapure lipopolysaccharide from the bacterium *Rhodobacter sphaeroides* (LPS-RS)) to inhibit the production of GP-induced cytokines [63]. Their study gave an in vivo evidence that the early stimulation of proinflammatory cytokines during EBOV infection is via the TLR4 pathway.

The RLRs are also host PRRs which are involved in the regulation of innate immune responses by recognizing the pathogen-specific 5′ di or triphosphate non-self RNA in bacteria and viruses, consequently leading to the transcription of IFN-β [64,65]. They can also recognize bacterial mRNA that is uncapped and is 5′ triphosphorylated in the cytosol [66]. He et al. demonstrated that EBOV VP24 inhibits both IFN-induced antiviral responses and type III IFN-γ1 gene expression by inhibiting the RIG-1 pathway responsible for the IRF3 activation [67], indicating the role played by RLRs during EBOV infection. Whereas, NLRs which also regulate the innate immune responses by triggering NF-κB signaling for expression of innate immune responses genes and hydrolyzing viral RNA using activated RNase, do so by recognizing 2′, 5′ -oligoadenylate synthetase type 2 (OAS2) in bacteria and viruses [65,68,69].

The primary role of DCs is to mediate cellular immune response (CD8^+^ T cells and CD4^+^ T cells) and humoral immune responses (B cells) [70,71,72], which are of great importance in developing vaccines. In the development of vaccines, DCs are targeted to elicit innate and acquired immune responses by capturing antigens or foreign material at their initial location in the peripheral tissues, processing and presenting antigens on major histocompatibility complex I and II (MHC I and II) [73]. DCs can also be used as adjuvants for DNA vaccines to elicit immune responses [28,52,74,75].

Meanwhile, macrophages are myeloid progenitor immune cells that are characterized by avid phagocytosis because they ingest and degrade dead cells, debris, and foreign material and orchestrate inflammatory processes in the body tissues [44]. They originated from either embryonic development or circulating monocytes [76] and are found all over the body in tissues by adhering to mucosal surfaces and can also be further classified based on their microenvironment [77]. Macrophages serve as the vital component of the innate immune system and also function as professional antigen-presenting cells [78,79]. Besides their role as APCs for the stimulation of specific cellular and humoral immune cells, macrophages also critically regulate the innate immune system by eliciting proinflammatory cytokines and chemokines such as interleukin-6 (IL-6) and tumor necrotic factor (TNF), as well as anti-inflammatory cytokines such as IL-10 [63]. Deficiency of macrophages in mice has been demonstrated to significantly reduce the protection ability of opsonizing antibodies, suggesting its crucial impact on the induction of immune response [80].

In the immune system, a relationship exists between DCs and macrophages. DCs stimulate autoimmune responses to induce specific T cells, that consequently leads to the proliferation of macrophages, which damages the tissue [79]. However, macrophages are involved in the homeostasis of tissues and repair, which helps to prevent tissue damage [79]. The DCs and macrophages connect innate immunity with adaptive immunity. DCs and macrophages are activated during infection for protection by recognizing pathogen-associated molecular patterns (PAMPs) via their PRRs [81]. Upon activation, the matured DCs migrate to the lymph nodes and display the processed peptides on their MHC I or II to trigger T cytotoxic cells (CD8^+^) or T helper cells (CD4^+^), respectively [82].

### 1.3. EBOV Infection and Immune Responses

Ebola virus causes hemorrhagic viral infection by primarily infecting the macrophages and the DCs upon contact with the mucous membrane, and replicate efficiently. Furthermore, the Ebola virus can impede interferon production in DCs, macrophages, and monocytes by protein VP24 and VP35 [83,84,85]. The EBOV glycoprotein (GP) enhances the entry of the Ebola virus to DCs/ macrophages by the presence of C-type lectin-like receptors (CLRs) present on the DCs [86,87]. As described elsewhere, EBOV can also evade the immune system by vitiating both the humoral and cellular adaptive immunity [84]. Although the mechanisms by which EBOV mitigate the host humoral and adaptive immune responses are poorly understood, the depletion of T-cells during EBOV infection has been hypothetically implicated with the deficient signaling events needed during the induction and maintaining the transition of T cells to memory cells and partial clearance of APCs [88]. Furthermore, Lubaki et al. demonstrated that the IFN-inhibiting domains (IIDs) in VP24 and VP35 also contribute to the depletion of the immune system by inhibiting the T cell receptor binding and are also responsible for deficient matured DC [89]. Lubaki et al. also recently revealed that the IIDs in VP24 and VP35 could vitiate humoral immune responses by inhibiting the human B cells differentiation and activation [90]. Whereas, the association of EBOV GP with DCs facilitates the ability of the EBOV GP to modify immune responses by modulating both innate and adaptive immune responses [82]. Interestingly, Groseth et al. demonstrated that although EBOV GP is involved in EBOV infection, EBOV GP alone is not sufficient to cause a lethal effect on the host [91].

The evasion of the immune system consequently affects the vascular system to cause coagulopathy, leading to shock, failure of circulation, bleeding, and death. Other complications are defective inflammations associated with mild secretion of IL-6 and TNFα and a very high level of secretion of IL-1, IL-10 [92], and flawed immune responses such as enormous apoptosis of T cells and the inhibition of the production of specific antibodies [84,93].

To further elucidate the relationship between EBOV GP and APCs, Lüdke et al. showed that a subset of DCs reduced significantly among patients that had acute EBOV in Guinea, while the survivors had activated CD16+ during recovery [94]. The study of Lüdke et al. further showed that EBOV primarily infects DCs, and patients still require DCs to fight and clear EBOV infection. Also, using a chimeric mouse characterized by a competent hematopoietic-immunity, the same authors demonstrated that EBOV primarily infects CD11b+ DCs in both the lymphoid tissues and non-lymphoid tissues which can lead to the depletion of CD8 and CD4 T cells [95]. Although there are reports that showed that some of DCs subsets, including CD141+ DCs, are not prone to viral infection by RAB15, which is expressed on CD141+ DCs and serves as a vesicle-trafficking protein [95,96], the population of DCs that are primarily infected by EBOV is enough for the modulation of both the innate and adaptive immune responses. Moreover, Silvin et al. showed that CD141+ DCs can still act as APCs for the regulation of adaptive immune responses by depending on the viral antigen from bystander cells [96].

Studies have demonstrated that EBOV infection can trigger macrophages to induce innate immune responses, such as inflammatory cytokines and chemokines (e.g., tumor necrosis factor, IL-6, IL-1β, etc.) [97]. EBOV GP is involved in the stimulation of both innate and adaptive immune responses. A study showed that immunization of mice with liposome-encapsulated irradiated Ebola virus induces immune response against Ebola virus via Ebola GP [98]. More recently, a group of scientists proved that Ebola GP can elicit an innate immune response, such as proinflammatory cytokines including IL-6, TNF-α, and anti-inflammatory cytokines and IL-10 alone without adjuvant, which depends solely on the internalization of the EBOV GP by macrophages [63]. They further elucidated that the efficacy of the current vaccine for Ebola virus largely depends on the innate immune response induced by EBOV GP through the toll-like receptor-4 (TLR4) pathway. Ayithan et al. also demonstrated that the induction of chemokines by EBOV GP is via the TLR4 pathway [99]. The role played by EBOV GP in the stimulation of immune response has thus been considered as a significant platform for generating a vaccine for EBOV infection [100].

## 2. EBOV GP: Bane or Benefit

The synthesis of 676-residue transmembrane of EBOV Glycoprotein (GP) results from the transcriptomic editing of the fourth of the eight (8) genes in the genome of EBOV [101,102]. The EBOV GP is responsible for targeting cell and virus entry by mediating receptor binding and membrane fusion [103]. GP is the only surface protein on EBOV, and it is cleaved by furin to produce disulphide-linked GP_1_ and GP_2_ subunits [104]. The endosomal entry of EBOV is by GP_1_, while the low pH membrane fusion is coordinated by GP_2_ using Neimann-Pick C1 protein (NPC1), and thus implicated as major pathogenic determinants for infection [101] and the main target for the development of a vaccine for Ebola virus [105].

**Figure 1:**
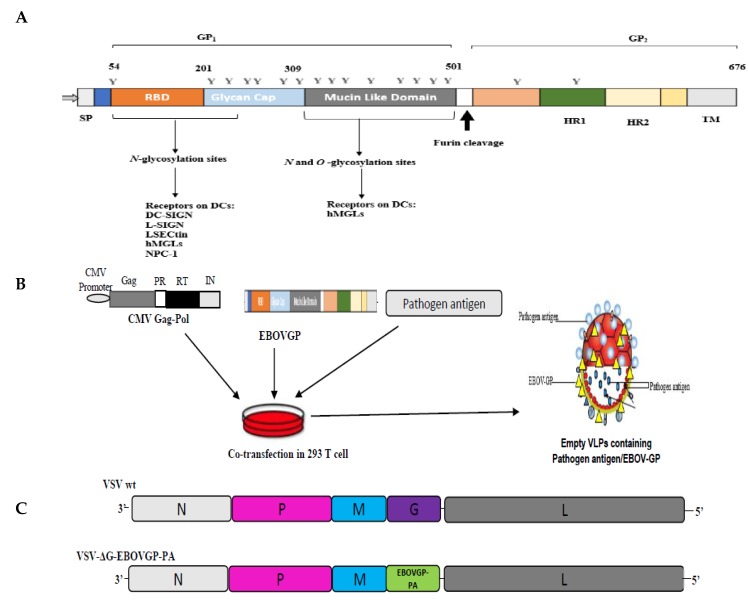
A schematic structure of (**A**) EBOV GP, indicating GP_1,2._ GP_1_ comprised of RBD, glycan cap, and MLD, while GP2 contains the HR1 and HR2. *Y* denotes the N-glycosylation sites. Receptors on DCs have an affinity with the N-linked glycans on GP_1_, indicating that the binding sites of EBOV GP with DCs are on the RBD, while the glycan cap contributes to its binding because of the presence of N-glycosylation sites [109]. The receptors on DCs for GP_1_ include DC-SIGN [110], L-SIGN, LSECtin [111], hMGLs [112,113], and NPC-1 [114]. Although N-glycosylation sites are present on the MLD, MLD is dispensable, and its absence contributes to more efficient cell entry of EBOV GP [115]. (**B**) Schematic diagram showing the incorporation of EBOV GP with a different pathogen antigen into VLPs [115]. (**C**) Schematic structure of vesicular stomatitis virus (VSV) with deleted glycoprotein and having EBOV GP with different pathogen antigen in the deleted G domain of VSV [116]. (N, nucleoprotein; M, matrix protein; L, RNA polymerase; G, glycoprotein; P, phosphoprotein)

As previously described, GP_1_ is a membrane surface protein that comprises three main subunits, including the base, composed of β sheets and Cys53, that may be responsible for the intermolecular bridge with Cys609 of GP_2_ subunit. The second subunit of GP_1_ is the head, located between the base and glycan cap. The glycan cap is the third subunit, characterized by the presence of N-linked glycans [101,106]. The recent description of GP_1_ revealed that EBOV GP has three subdomains, including the receptor-binding domain (RBD) (approximately 149-residue), mucin-like domain (MLD), and the glycan cap (approximately 108-residue) [107,108] (Figure 1A). The MLD is also another highly glycosylated domain on EBOV GP. Unlike the glycan cap, which has only N-linked glycans, MLD has both N-linked glycans and O-linked glycans [108]. Lennemann et al. showed that the removal of all the 15 N-glycosylation of EBOV GP using site-directed mutagenesis significantly increases the pseudovirion transduction of EBOV in Vero cells. However, the removal of the N-glycosylation also favors the recognition of the EBOV GP by antibodies, resulting in the production of neutralizing antibodies [108].

The MLD is found on the variable region of GP_1_ (C-terminal) and increases the permeability of EBOV into the blood vessels, and also masks the cell from the innate immune response by obstructing access to the epitope of GP [117,118]. MLD can also shield the cellular surface protein sterically, causing cell damage, leakage of an explanted blood vessel, rounding and detachment of cell, and loss of physiological functions [119,120,121]. A study showed that the MLD blocks access to the surface MHC I and II, which leads to decrease in CD 8^+^ cells and consequently leads to cell rounding (cytopathic effect), while the removal of MLD uncovers the epitope of GP to induce neutralizing antibodies [120,122]. Another study also checked for the impact of EBOV GP without MLD (EBOV GP ΔMLD) on the stimulation of anti-GP and neutralizing antibodies; this study revealed that EBOV GP ΔMLD elicits more anti-EBOV GP antibody than EBOV GP VLP, with moderate stimulation of neutralizing antibodies [107], indicating that MLD is dispensable for EBOV attachment. Our study also showed that the removal of MLD from EBOV facilitates the cell entry efficiently more than the wild type; however, EBOV GP wild-type stimulated NFκB than EBOV GP with deleted MLD [115]. Moreover, in the development of drugs for EBOV, EBOV GP is an important target to be considered. A study showed that the inhibition of GP_1_ binding by toremifene (an antiviral drug against EBOV) could lead to the premature release of GP_2_, and thus prevent fusion of the endosome membranes and the virus [123].

On the other hand, GP_2_ contains the heptad repeated regions 1 and 2 (HR1 and HR2) and internal fusion loop (FL) lacking a free N-terminus (511–556-residue), which display a hydrophobic fusion peptide by utilizing an antiparallel β (Figure 1) [101]. During proteolytic cleavage and endosomal binding of GP_1_, GP_2_ undergoes a conformational rearrangement which exposes FL for fusion [124]. Lee et al. recently presented the membrane-proximal external region (MPER) as the missing part of GP_2_ that is connected to the transmembrane using NMR and EPR spectroscopy. They further showed that MPER consist of ‘helix-turn-helix architecture.’ Lee et al. also revealed the role MPER played by mutating the aromatic neighboring, and the results revealed that the MPER region interacts with EBOV FL through aromatic residues and the mutation of these aromatic residues decreases the fusion and viral entry of EBOV [124]. Although GP_2_ contains two N-glycosylation sites, Asn^563^ and Asn^618^, Wang et al. demonstrated that EBOV GP expression does not depend on GP_2_. However, their study highlights the functions of N-glycosylation sites on GP_2_, which includes regulation of GP processing, oligomerization, demannosylation, conformation, and facilitation of the incorporation of EBOV-like particles and pseudovirions of HIV type 1 (HIV-1) for the determination of viral transduction efficiency [125].

Moreover, Lee et al. described a 364-residue of non-structural secreted glycoprotein (pre-sGP) that contains the gene product of EBOV, which is also emitted during the transcriptional editing of the fourth gene of EBOV [101,102] and results from the unedited mRNA [126]. Briefly, sGP is produced from the post-translational cleavage of pre-sGP at the C-terminus by furin—a cellular protease. The proteolytic cleavage produces Δ-peptide and sGP, and while we know little about the receptors and role played by Δ-peptide during EBOV infection, a study has demonstrated that Δ-peptide competes with the binding of EBOV GP when interacting with the host permissive cells for EBOV [127]. On the other hand, sGP forms homodimer by the linking of its monomers at residue Cys53 and Cys306 [128] and just like the GP_1_, sGP is N-linked glycosylated [126]. Several studies have been done to highlight the role played by sGP during EBOV infection and their effect on immune responses (see review [126]). Research has also recently demonstrated the functions of sGP during the pathogenesis of EBOV. In their study, Wahl-Jensen et al. showed that sGP could not induce production macrophages [129]. Also, Monath et al. showed that the construction of rVSVΔG-ZEBOV-GP lacking sGP produces more neutralizing antibodies against EBOV GP [130], because sGP can vitiate the neutralizing of EBOV GP by acting as a decoy for the antibody responsible for the neutralization of EBOV GP [126,130,131,132].

The GP and sGP are identical in the N-terminal, with 295 amino acids, but are different at the C-terminal. At the C-terminal, sGP has about 65 amino acids, while GP has 381 amino acids, including the mucin-like domain [107,133,134,135]. The similarity in the N-terminal of GP and sGP has been suggested to be responsible for a term called antigenic subversion by sGP, which prevents an immune response to GP by diverting it away. Mohan et al. demonstrated in mice immunized with GP_1,2_ and sGP, that sGP competes efficiently for anti-GP_1,2_ antibodies by refocusing the host antibody response to the membrane-bound, thus underscoring the robust immune response responsible for clearing Ebola virus in the system [136]. Therefore, in developing VLPs for EBOV, the GP_1,2_ must be essential components because of their ability to induce immune response, while sGP might not be included in the EBOV VLP as it has low immunogenicity [129].

Nonetheless, EBOV GP has some right sides in mediating immunity. The EBOV GP can facilitate the maturation of DCs and activates T cells, as well as B cells [89], and can also induce changes in secondary target cells [105]. EBOV GP can upregulate the expression of costimulatory molecules in bone marrow-derived macrophages (BMDM), suggesting its capability to enhance APC stimulatory capacity, which is very important for the induction of potent antigen-specific adaptive immunity [63]. The recent development of Ebola vaccine (rVSV-EBOV Vaccine) is solely dependent on the immunogenicity of EBOV GP. The rVSV-EBOV vaccine was constructed using a recombinant form of vesicular stomatitis virus expressing the EBOV-glycoprotein (GP) on the surface (rVSV/ZEBOV-GP) [137]. Agnandji et al. conducted the phase 1 clinical trial of rVSV/ZEBOV-GP vaccine and reported that this vaccine could induce stable neutralizing antibodies against EBOV GP with a very mild side effect, such as fever [138]. The induction of the neutralizing antibodies is of no doubt associated with the targeting of DCs/macrophages by EBOV GP. This claim is in line with the study done by Marzi et al., which indicated the importance of antibodies for the protection against Ebola virus using a non-human primate [139]. Aside from the utilization of Ebola GP with rVSV for the development of a vaccine against Ebola virus, another study has shown that EBOV GP expressed on adenoviruses virus-like particles (VLPs) also stimulates immune responses [140]. Takada et al. reviewed that the ability of EBOV GP to induce both innate and adaptive immune responses, which could be via complement antibody-dependent enhancement (ADE) (see extensive review in [141]). Therefore, in this review, we will further describe the use of EBOV GP to induce immune response in the subsequent paragraphs.

## 3. The Interaction Between EBOV GP and DCs/Macrophages Can Induce Robust Innate and Adaptive Immune Responses

During exposure of EBOV to the susceptible cell, the main first line of defense is the innate immune response. The innate immune response is not specific, unlike the adaptive immune response, and is active during the critical hour of infection before the recruitment of adaptive immune responses. Phagocytic cells regulate innate immune responses by inducing inflammatory cytokines, and recognizing conserved features among many pathogens called pathogen-associated immunostimulants [142].

EBOV GP has also been investigated to induce inflammatory cytokines and chemokines, including interleukin (IL)-6, tumor necrosis factor (TNF)-α and IL-1β, interferon (IFN)-γ, IL-2, IL-5, IL-4, IL-12 p20, and IL-10, among others [97], through the Toll-like receptor (TLR)-4 pathway like many other GPs [63]. DCs and macrophages express TLRs, which is essential in the activation of MyD88-dependent and independent signaling pathways and consequently leads to the activation of transcription factor interferon regulatory factor 3/7 (IRF3/7), nuclear factor κB (NF-κB), and activator protein-1 (AP-1) to induce downstream genes [82].

Moreover, Lai et al. showed that immunization with EBOV GP without adjuvants can stimulate a significantly high amount of innate immune response. Lai et al. (2017) [63] further established that EBOV GP can induce a robust innate immune response even after 2 h of treatment, which was built upon the findings of Henao-Restrepo et al. (2015), who revealed that the rVSV-EBOV GP vaccine candidate induces innate immune response within six (6) days of immunization [143]. Furthermore, in contrast to other pathogens with recognizable PAMPS by the PRRs on the monocytes, EBOV GP causes the release of cytokine and chemokines in a manner leading to the recruitment of more DCs and macrophages to the site of infection [82,86].

EBOV GP also plays an essential role in mediating the interaction between innate and adaptive immune response. Although there are studies that have demonstrated that EBOV GP could impair the ability of CD8^+^ to recognize peptide on the MHC Class I [120], analysis from a 2013–2016 outbreak on the induction of CD8^+^ T cells revealed that about 40% of the populace examined stimulated CD8^+^ T cells by EBOV GP [144]. A comprehensive study on the cellular immune response by EBOV infection showed that CD8^+^ T cell is the predominantly induced T cell, but is relatively low [145]. However, the usage of adjuvant with EBOV GP can produce CD8^+^ T cells that are enough to protect against lethal Ebola virus challenge [146]. Generally, EBOV GP can trigger CD8^+^ T cell and CD4^+^ T cell by interacting with the DCs and macrophages. Thus, EBOV GP can be used to recruit more T cells. Targeting antigens towards DC-specific endocytic receptor, together with other relevant antibodies or ligands, can elicit durable and robust T cell responses against viral pathogens [31].

## 4. EBOV GP’s Affinity for Dendritic Cells and Macrophages in Ebola Virus Infection: An Insight for Vaccine Development

EBOV infects the macrophages and DCs by binding its GP with the CLRs on DCs/macrophages [86,87]. C-type lectins (CLRs) present on DCs interact with N- and O-linked glycans on GP_1_ (RBD, MLD, and glycan cap) (Figure 1) to facilitate viral entry. Unlike HIV-1 GP, EBOV GP has a variety of receptors on DCs and macrophages, making them an efficient and better stimulator of antigen-presenting cells. There are four members of CLECs that have been identified as Ebola GP receptors, namely, CLEC4G/LSECtin, dendritic cell-specific ICAM-3-grabbing non-integrin (DC-SIGN), liver/lymph node-specific ICAM-3 grabbing non-integrin (L-SIGN), asialoglycoprotein receptor 1 (ASGPRI) and human macrophage galactose- and acetylgalactosamine-specific C-type lectin (hMGL) [147,148]. CLECs are on the liver, alveolar macrophages, and epithelial cells [117,149,150]. Besides, the expressions of Nieman-Pick C1 (NPC1), integrin αV, and Mer have been reported to be essential for the infection of macrophages by EBOV GP. Although, some other receptors have also been previously reported for EBOV GP, including TAM receptor tyrosine kinases (Axl and Tyro3), T cell immunoglobulin and mucin domain (TIM proteins) [151], recent findings have demonstrated that TAM and TIM do not contribute to the EBOV GP-driven transduction of macrophages [152]. We therefore describe the three (3) major receptors on the DCs specific for EBOV GP below:

DC-SIGN: These are type II membrane protein and are expressed primarily on immature DCs [110]. DC-SIGN is involved in the initial mediation of immune responses by coordinating the DC interaction with T-lymphocytes and endothelial cells [110]. Other viruses such as measles [153], HIV [154,155], influenza virus [156], and HPV L1 [157] have specific receptors for DC-SIGN, which also help to internalize the virus into the DC for processing. The virulence of different species of Ebola virus, ranging from Reston Ebola Virus (REBOV) to Zaire Ebola Virus, depends on the differences in the N-glycan composition of their glycoprotein [158,159]. Thus, the large proportion of the high mannose N-glycans allows EBOV GP to interact with DC-SIGN and further leads to the induction of immune responses. L-SIGN, a homolog of DC-SIGN expressed on the endothelial cells in the placental villi, lymph node sinuses, and liver also has high mannose N-glycans for binding with EBOV GP. Development of an efficient vaccine depends on the antigenic or virulent factor of the invading pathogen; thus, studies for development of a DC-targeting vaccine can employ the modification of the N-glycans that target DC-SIGN(R) [160].

LSECtin: LSECtin, which is also known as CLEC4G, also mediates EBOV GP–DCs interaction to stimulate inflammatory responses. Liver and lymph nodes, sinusoidal endothelial cells express LSECtin [161], and Domínguez-Soto has also reported the expression of LSECtin in DCs and macrophages [162]. LSECtin also plays a vital role in the pathogenicity of the EBOV by serving as a receptor for GP_1_ for EBOV internalization [105,156,158]. Zhao et al. demonstrated that LSECtin can induce TNF-α and IL-6 production in DCs, suggesting that LSECtin can aid GP in inducing inflammatory responses [109]. In contrast to DC-SIGN and other glycan-binding receptors, the antibody-induced internalization by LSECtin on myeloid cells is not in clathrin-mediated endocytosis, but could aid the antigen capturing and presentation by DCs and macrophages [111,163]. Unlike most lectins, LSECtin does not interact with many viruses, but has a strong affinity for EBOLA GP and not HIV-1 GP. Gramberg et al. (2008) also showed that LSECtin and DC-SIGN act differently in the ways they capture pathogen, and even in the lectin biological functions [163].

hMGLs: Human macrophage galactose-type C-type lectins (hMGLs, CD301) are also transmembrane II proteins and play a critical role in the pathogenesis of EBOV. They are expressed on the DCs and macrophages and enhance adhesion of cells, internalization, and hematopoiesis [113]. The hMGLs have two homologs of MGL: MGL 1 (CD301a) and MGL 2 (CD301b) [112]; however, MGL 1 and MGL 2 have an affinity for Lewis trisaccharide (Galβ1–4[Fucα1–3]GlcNAc) and N-acetylgalactosamine, respectively [164]. hMGLs expressed on the monocyte-derived immature dendritic cells (MDDCs) and macrophages function as an endocytic receptor for galactosylated GP antigens [165]. In the affinity of hMGLs and EBOV GP, the highly glycosylated mucin-like-domain must be present for efficient interaction [165]. Usami et al. demonstrated that EBOV GP_2_ interacts with hMGLs of macrophages and DCs via the N-acetylgalactosamine for cell entry of the virus and to initiate infection [166].

In all, the N-glycan moieties and N-acetylgalactosamine present on the EBOV GP_1_ are essential features for the binding to the CLRs on the macrophages and DCs. Further modification of the binding sites on EBOV GP can influence the binding efficiency of EBOV with lectin receptors and other cellular factors to facilitate the activation of APCs.

Moreover, another possible receptor on DCs has been identified to have an affinity for EBOV GP. This receptor is known to be a hydrophobic Neimann-Pick C1 (NPC-1) receptor-binding pocket. Bornholdt et al. showed that EBOV GP could bind with the endosomal (NPC-1) receptor on DCs, initially by interacting electrostatically with the NPC-1 by the hydrophilic crest on GP_1_, while hydrophobic trough exposure on GP_1_ facilitates specific interactions due to their ability to migrate to lymph nodes where they can interact with DCs [114]. Their results revealed that mutation of the GP_1_ to block the hydrophilic and hydrophobic sites on the GP_1_ inhibit infectivity and binding of GP_1_ with NPC-1. They also demonstrated that the observed interaction leads to the stimulation of monoclonal neutralizing antibodies. This important finding is significant in developing a DC-targeting vaccine using EBOV GP. The hydrophilic crest and the hydrophobic trough of EBOV GP_1_ can be used heterogeneously with other viral protein to direct these peptides to DCs.

## 5. Ebola GP-Targeting DCs Can Facilitate Immune Responses for an Antiviral Vaccine Approach

An essential feature of antigenic agents is the ability to induce innate and adaptive immune response, as well as humoral and cellular immune responses. It is interesting to find out that the affinity of EBOV GP with DCs and macrophages can not only induce an adaptive immune response by recruitment of DCs/macrophages and facilitation of the maturation of DCs /macrophages [63], but can also induce innate immune responses which can serve as protection against other viruses. In the development of Ebola virus vaccine, EBOV GP has been shown to play a significant role, as both the VLP and vector-based approach depend so much of Ebola virus. [167]. Briefly, we will elucidate how EBOV GP can be used to stimulate DCs and macrophages for vaccine production.

### 5.1. EBOV GP-Coated Virus-Like Particle Vaccine Approach (VLP)

EBOV GP can be incorporated into VLPs to enhance the stimulation of DCs and macrophages, which, in turn, function in inducing adaptive immunity and interact with innate immune cells (Figure 1B) [168]. The efficiency of VLPs is undoubtful as it has succeeded in the induction of immune responses against several viruses, such as Rotavirus, among others [169]. Considering the high immunological characteristics of EBOV GP, it was co-expressed with matrix protein (VP40) to produce VLP. Warfield et al. showed that EBOV GP VLPs are immunogenic by facilitating the maturation of macrophages and DCs to induce the secretion of IL-10, IL-6, tumor necrosis factor α, and macrophage inflammatory protein (MIP)-1α [169]. This immunogenic property of EBOV GP VLP suggests that EBOV GP VLP is a promising tool for the development of a vaccine for Ebola. Moreover, EBOV GP is relevant as a tool to develop a universal vaccine against other viruses due to its ability to induce innate immune responses. There are ongoing clinical trials to test the efficiency of EBOV GP VLP vaccine against EBOV [140]. A recent study showed that EBOV GP VLP (consisting of VP40, NP, and GP) enhances the stimulation of DCs and macrophages [170]. Also, Venezuelan Equine Encephalitis (VEE), virus-like replicon particles with the replacement of VEE virus structural genes by EBOV GP or NP, has been demonstrated to have full protection against Ebola virus challenge [171]. Interestingly, our recent study showed that the incorporation of EBOV GP into the HIV VLP induces a more effective immune response against HIV-1 [115]. We showed that the presence of EBOV GP enhances the ability of the HIV VLP to target MDMs and MDDCs. Also, we revealed that EBOV GP-pseudotyped HIV VLP induces a significantly stronger humoral immune response than that of HIV VLP alone. Furthermore, macrophages inflammatory cytokines (MIP-1α) is significantly induced in the spleen by EBOV GP-pseudotyped HIV VLP more than HIV VLPs [115]. The heterogenic induction of immune responses by EBOV GP suggests that the immunogenicity of EBOV GP is not only beneficial in the development of a vaccine for EBOV, but can also be used to develop a vaccine for some other infectious diseases. Also, Wong et al. incorporated HA of H5N1 into the VSVΔG-ZGP (a previously described vaccine for EBOV) to form a bivalent vaccine, VSVΔG-HA-ZGP, which protects against both EBOV and H5N1 lethal challenge [116]. In this study, the presence of EBOV GP targeted the peptides of influenza HA to DCs/macrophages, which processed and presented the HA peptide, on MHC I or II for the eliciting of T-cells specific for influenza H5. [116]. Also, Chahal et al. also demonstrated that an adjuvant free dendrimer nanoparticle vaccine has broad protection against Ebola virus, influenza H1N1, and *Toxoplasma gondii* [172]. The eliciting of immune responses by this vaccine depends on dendrimer nanoparticle vaccine platform in which a dendrimer nanoparticle is encapsulated with mRNA replicons to generate specific CD8^+^ T cell antibody responses.

### 5.2. EBOV GP and Vector-Based Vaccine

Different vector-based vaccines are also an effective platform for the development of a vaccine for EBOV, ranging from vaccinia virus-based vaccines expressing ZEBOV GP, VP24, VP35, and VP40 [173], adenovirus-based vaccines having ZEBOV GP (AdHu5-ZGP), and combination with ZEBOV NP, SEBOV GP, and ICEBOV GP as a DNA vaccine [167] and Vesicular stomatitis virus (VSV)-based candidate vaccines [167,174]. The use of recombinant VSV to develop a candidate vaccine induces a strong humoral and cellular immune response and gives 100% protection in an animal model [167,174]. VSV used as an expressing vector for foreign proteins has a small amenable genome feature for genetic manipulation; thus, it is suitable for vaccine development [174]. Furthermore, the efficacy of chimpanzee adenovirus three vectored vaccine expressing EBOV GP has been demonstrated both in monovalent and bivalent forms in clinical trials in the UK, Europe, the USA, Nigeria, and Mali [140,175,176]. Also, in a clinical trial, a modified vaccinia Ankara vectored quadrivalent vaccine consisting of GPs of EBOV, Sudan Ebola virus, and Marburg virus and NP from the Tai forest strain boosted the humoral and cellular adaptive immune system, including neutralizing antibodies [170]. And recently, Zhu et al. showed that recombinant human adenovirus-vectored vaccine (rAd5—vectored vaccine) encoding GP is safe, with very high immunogenicity among adults in Sierra Leone and China with the requirement of high dose [177]. The efficacy of VSV–EBOV has been demonstrated in cynomolgus macaques to give 100% protection [21]. Also, human phase 1–3 trials have revealed the effectiveness of VSV–EBOV GP in inducing an immune response against EBOV [100,117]. Thus, EBOV GP infused with a specific viral antigenic protein can be incorporated into VSV as a vector-based vaccine (Figure 1C) to induce stronger and more robust immune responses against the specific virus [116].

## 6. Conclusions

Dendritic cell-targeted vaccines and EBOV GP-based vaccines are very potent, long-lasting, durable, and safe vaccines [31]. Based on the ability of EBOV GP to induce the stimulation of DCs and macrophages for the modulation of the cellular and humoral adaptive immune responses, EBOV GP can be used for the development of a DC-targeting vaccine approach and used as a natural adjuvant to elicit robust acquired immune responses. Since adjuvants are substances which can either be biological (microbial products, saponins, cytokines, and liposomes), chemical (mineral salts, polymers, and emulsions), or even particles (microparticles and nanoparticles) that can aid in the production of robust and stronger immune responses when combined with a specific antigen more than using the antigen alone [178], we propose that EBOV GP can also serve as natural adjuvant with no adverse effects. As mentioned above, since EBOV GP can target different peptides to the DCs, it can thus aid in the production of robust immune responses for specific antigens. Few pieces of research, as earlier mentioned, support this claim. Therefore, further investigations are recommended for the usage of EBOV GP as a substance to direct specific antigens to DCs for induction of stronger immune responses than what the peptide can produce alone.

**Figure 2:**
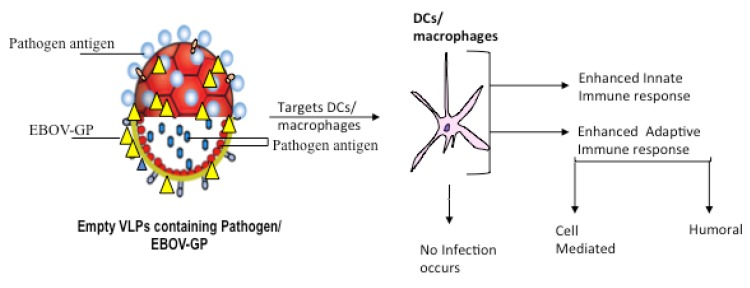
Schematic diagram of the incorporation or infusing pathogen antigen on EBOV GP to target DCs for the induction of immune responses. Using the technology of EBOV GP VLP immunological basis, it is possible to incorporate EBOV VLPs with other viral antigens to induce an efficient humoral and adaptive cellular immune responses.

Likewise, since EBOV GP_1,2_ also play an essential role in the stimulation of innate immune responses, this viral glycoprotein can be used to stimulate the induction of inflammatory cytokines. Using the technology of EBOV GP VLP immunological basis, it is possible to incorporate EBOV GP VLPs with other viral antigens to induce strong humoral and cellular adaptive immune responses (Figure 2). Since studies have established the immunogenic properties of EBOV VLPs and successful development of EBOV VLP vaccines and DCs-targeted vaccines, these immunological approaches can be further researched to develop a vaccine for other viruses, including HIV, influenza, Zika viruses, and other epidemic and pandemic viral infections. Also, EBOV GP can be fused with other viral proteins and inserted in the deleted G domain of VSV as a vector-based vaccine to induce stronger immune responses. Having shown the potentials of EBOV GP in inducing robust immune responses by directing specific antigens of peptides to DCs, further studies are recommended using EBOV GP to develop innate and adaptive immune responses to any desired pathogen. Furthermore, it is expedient to investigate possible limitations and variations to this technology.

## Figures and Tables

**Table 1:** Vaccine development strategies for selected viral infections.

S/N	Viral Infection	Vaccine Development Strategy	Route of Administration	Vaccine Status	References
1	Yellow fever	Attenuated virus	Subcutaneous	Available in circulation	[5]
2	Influenza	Live attenuated virus, inactivated virus, recombinant influenza vaccine, VLP	Intramuscular, intranasal, intradermal, subcutaneous	Available in circulation VLP and DNA vaccine not in circulation (needs development of a novel universal vaccine)	[6,7,8]
3.	Poliovirus	Live attenuated vaccine	Oral	Available in circulation	[9]
4.	Measles, mumps and rubella	Live attenuated vaccine	Subcutaneous	Available in circulation	[10]
5	Human papilloma virus	VLP, inactivated vaccine	Intramuscular, oral	Monovalent, bivalent, tetravalent, nonavalent vaccines available in circulation	[11,12]
6	Hepatitis B virus	Live inactivated, recombinant DNA	Intramuscular	Available in circulation (it gives short-term protection; issue raised concerning its safety)	[13,14,15]
7	Varicella	Weakened live virus or attenuated virus	Subcutaneous, intramuscular	Available in circulation	[16]
8	Rotavirus	Live attenuated, VLP	Oral, intranasal	Available in circulation; VLP not in circulation	[17,18]
9	HIV	VLP, DNA vaccine	Subcutaneous, intramuscular	Not in circulation (development in progress)	[19,20]
10	EBOV	Live attenuated, VLP	Intramuscular	Available but not yet in circulation (phase trial in progress)	[21,22]
11	Lassa virus	VLP, live attenuated virus, DNA vaccine	Intradermal	No available vaccine	[23,24,25]

Note: EBOV, Ebola virus; VLP, virus-like particle; HIV, human immunodeficiency virus.

**Table 2:** Development of vaccine using a dendritic cell (DC)-targeted approach in the selected viruses.

S/N.	Vaccine	Immune Responses Induced	Vaccine Development Strategy	Route of Administration	DC-Targeting Substance	Protection Percentage	Ref
1	Cancer vaccine	Cellular immune response and humoral responses	DNA vaccine	Not applicable	IFN-α	Not applicable	[38,39]
			DNA vaccine	Subcutaneous	Liposome and melanoma	80–100%	[39]
2	Yellow fever	Innate immune responses (proinflammatory cytokines interleukin (IL)-12p40, IL-6, and interferon-α), adaptive immune responses (T helper cell (Th)1/Th2 cytokine profile and antigen-specific CD8^+^ T cell)	Live attenuated vaccine	Subcutaneous		Not applicable	[30]
3	Adenovirus	Cytolytic T lymphocyte cells	Recombinant vaccine	Not applicable	Recombinant single-chain (sc) mAb Fv fragments	Not applicable	[29]
4	HIV	IFN-γ, CD4^+^, and CD8^+^ T cell	Recombinant vaccinia virus (DNA vaccine)	Intranasal	Recombinant single-chain (sc) mAb Fv fragments (scFv) HIV gagp41-scFv	100%	[40]
5	Influenza A	Cytotoxic CD8^+^T, cell CD4^+^ Th1, IgG2a antibodies	DNA vaccines	Intradermal	Xcl1-hemagglutinin (HA)	100%	[41]
			DNA vaccine	Intravenous tail injection and electroporation	Xcl1-HA or Xcl2-HA	90%	[42]
6	West Nile Virus vaccine	Humoral and T-cell responses	DNA vaccine (immunodominant vaccinia B8R gene)	Intravenous injection	Rabies glycoprotein (GP) fused to protamine residue (RVG-P)	80%	[43]
